# Mechanistic Insights into Delayed Bruise Clearance after Skin Booster Injection: Role of Blood–Polymer Complexation

**DOI:** 10.34133/bmr.0445

**Published:** 2026-11-12

**Authors:** Soonjong Roh, So-Jin Park, Seongryeol Ye, Youngmee Jung, Chanjong Lee, Olha Shestakova, Bilal J. Malik, Jimyung Seo, Hojun Kim, Jin Yoo

**Affiliations:** ^1^ Center for Biomaterials, Korea Institute of Science and Technology (KIST), Seoul 02792, Republic of Korea.; ^2^ Department of Chemical Engineering, Chung-Ang University, Seoul 06974, Republic of Korea.; ^3^ Department of Applied Bioengineering, Graduate School of Convergence Science and Technology, Seoul National University, Seoul 08826, Republic of Korea.; ^4^ School of Electrical and Electronic Engineering, YU-KIST Institute, Yonsei University, Seoul 03722, Republic of Korea.; ^5^ Division of Bio-Medical Science & Technology, KIST School, University of Science and Technology, Seoul 02792, Republic of Korea.; ^6^ 4-ever Clinic Myeongdong, Seoul 04534, Republic of Korea.; ^7^ Dexlevo Inc., Medical Research Department, Seoul 06093, Republic of Korea.; ^8^ Sculpted by Doctors Clinic, Bolton, UK.; ^9^ Galleria Dermatology Clinic, Seoul 06325, Republic of Korea.; ^10^ Center for Advanced Biomolecular Recognition, Korea Institute of Science and Technology (KIST), Seoul 02792, Republic of Korea.

## Abstract

Cutaneous injection of polycaprolactone-based skin boosters has emerged as a promising approach for facial rejuvenation, leveraging their long-lasting effects and ability to stimulate collagen regeneration. Despite clinical success, complications such as delayed bruise clearance remain poorly understood mechanistically. Here, we establish a controlled murine bruise model to investigate the impact of blood–polymer interaction and injection sequence on bruise persistence. To model distinct blood–polymer contact sequences, blood was administered before or after the polymer, while premixed blood–polymer formulations served as comparison groups. Bruise appearance was monitored and complemented by histological, spectroscopic, and colorimetric analyses of blood persistence, inflammatory responses, scaffold structure, and material degradation. Comprehensive in vitro rheological and structural assays confirmed rapid temperature-triggered sol-to-network transition and scaffold formation. In vivo, sequential blood–polymer delivery markedly exacerbated bruising, scaffold remodeling, and inflammation compared to coinjection controls. The sequential groups maintained higher late-stage red blood cell density and showed slower discoloration, supporting physical restriction of blood dispersion and clearance at the injection site. Cryogenic scanning electron microscopy and Raman spectroscopy revealed blood retention within remodeled polymer scaffolds as a key mechanism. This study highlights critical injection parameters and provides mechanistic insight into bruise pathophysiology, offering a foundation for safer, optimized skin booster therapies.

## Introduction

Cutaneous injection of skin boosters and dermal fillers has become increasingly common in aesthetic practice, providing minimally invasive approaches for facial rejuvenation and skin quality enhancement [1–4]. Nevertheless, early adverse events such as hematoma formation and bruising remain important procedural concerns [5,6]. These persistent discolorations and localized swellings can necessitate social downtime and negatively influence patients’ willingness to undergo repeated procedures. These manifestations are particularly problematic in highly vascularized facial regions, where resolution may take weeks to months, thereby directly impacting overall treatment satisfaction.

Among available injectables, polycaprolactone (PCL)-based skin boosters have garnered considerable interest due to their durability and capacity to induce neocollagenesis through in situ scaffold formation [7–9]. Recently, a liquid-type PCL (GOURI, Dexlevo Inc.) has been introduced to address some of the limitations associated with conventional microparticle-based dermal fillers [8]. Conventional PCL fillers typically consist of microspheres (20 to 50 μm in diameter) suspended in an aqueous carboxymethyl cellulose gel carrier [10]. While effective, this particulate suspension is associated with specific procedural and safety challenges, including needle occlusion, nonhomogeneous distribution, and the risk of delayed-onset granulomatous reactions, as well as vascular complications [11–16]. A key distinction of liquid-type PCL from traditional particulate-based injectables is the absence of preformed solid particles. This characteristic may facilitate distribution within the injection layer and allow the formulation to be administered using devices such as needle-free injectors [17] to target more superficial dermal layers. The absence of solid microparticles may also reduce particle aggregation and particulate-associated foreign-body responses [18].

However, the absence of particulate material does not eliminate adverse events associated with the injection procedure itself. In clinical practice, vascular injury and blood extravasation may occur before, during, or after polymer delivery, depending on the timing of needle passage and product administration. Consequently, extravasated blood may encounter the PCL formulation while it remains in the liquid state, during scaffold formation, or after a scaffold has already formed. Although bruising is conventionally attributed primarily to mechanical vascular injury [19,20], this explanation does not fully account for delayed bruise resolution or interindividual variability. Previous clinical observations have therefore suggested that scaffold-associated retention of extravasated blood components may also contribute to prolonged bruise-like discoloration [17]. However, the relative contribution of this material-dependent effect remains unclear. In particular, how the timing of blood exposure relative to scaffold formation influences scaffold architecture, blood retention, and hemoglobin processing remains poorly understood.

To address these unresolved questions, the present study systematically investigates the interaction between PCL-based skin boosters and blood components using controlled in vitro and in vivo approaches. We focus on how the temporal sequence of vascular injury and polymer injection affects the microenvironment of extravasated blood. We hypothesize that sequential blood–PCL exposure may promote local retention of blood components, resulting in slower bruise resolution and altered hemoglobin processing (Fig. 1). Here, “delayed bruise resolution” refers to a slower reduction in visible bruising relative to the control condition within the 10-day observation period, rather than clinically persistent bruising lasting for weeks to months. By integrating rheological characterization, cryo-scanning electron microscopy/energy-dispersive x-ray spectroscopy (cryo-SEM/EDX) analyses, cytocompatibility assays, and a murine injection model, this study investigates scaffold-associated blood retention as a potential material-dependent contributor to delayed bruise resolution and examines how the temporal sequence of blood–polymer contact influences this process. These findings may provide a basis for optimizing injection strategies and developing skin booster formulations aimed at reducing prolonged postinjection discoloration.

**Fig. 1.**
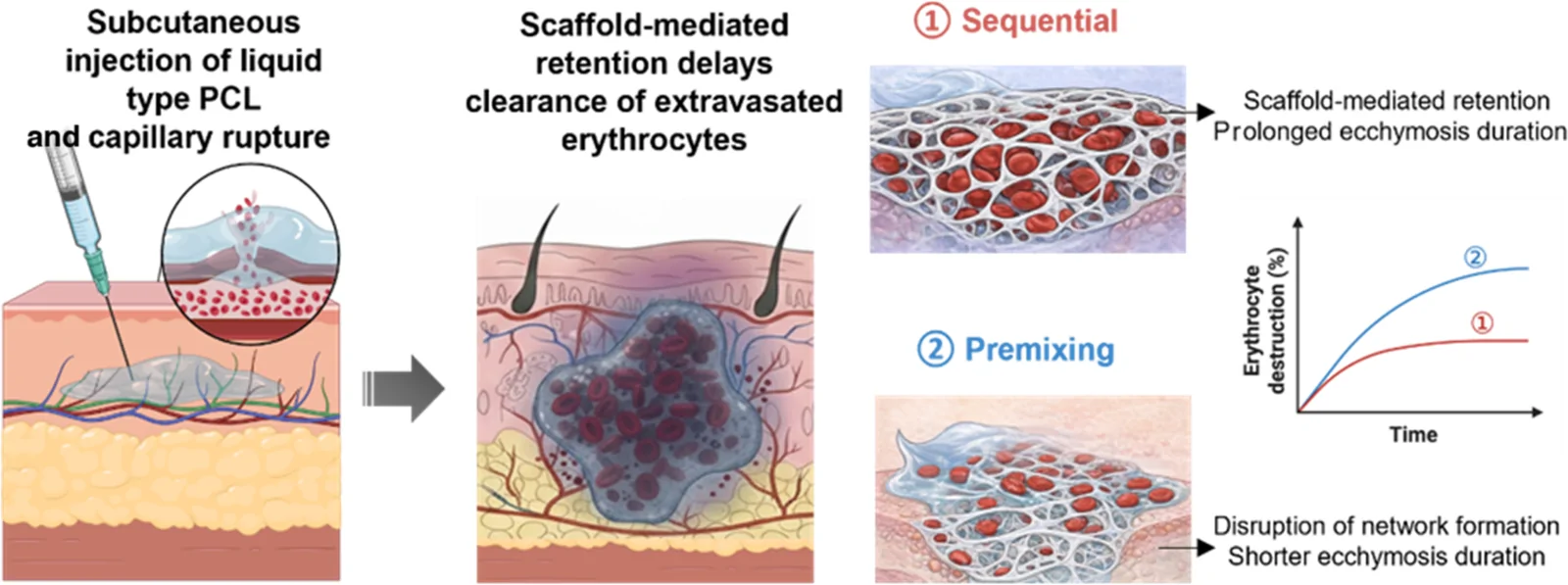
Schematic illustration of the proposed effects of red blood cell (RBC)–scaffold interactions on erythrocyte retention and bruise resolution.

## Materials and Methods

### Materials

Liquid-type PCL (GOURI, Dexlevo Inc., South Korea) was provided by Dexlevo Inc., and all other reagents were obtained from commercial sources as indicated in the main text.

### Cryo-SEM

Liquid-type PCL was freeze-fixed between 2 aluminum pans and subsequently processed in a freeze-fracture unit under vacuum in a high-pressure freezing device (ZEISS, Crossbeam 550 FIB-SEM).

### Mechanical testing of injectability

A universal testing machine (UTM, Instron Model 5966) was employed to evaluate the injectability of liquid PCL [21]. The relationship between injection force and displacement was determined using a 31G, 8-mm needle. A custom-designed syringe holder was mounted on the UTM, and syringes loaded with liquid-type PCL formulation were firmly fixed in place. The plunger was linked to the moving crosshead, which was advanced at a constant rate of 6 mm/min. Injection force was continuously monitored from the onset of injection until the PCL scaffold was completely expelled.

### Viscoelastic properties

The viscoelastic properties of the PCL scaffold were measured using a rheometer (Anton Paar, MCR102) equipped with a cross-hatched parallel plate geometry (25 mm diameter) at a gap of 0.3 mm. Before oscillatory measurements, the samples were allowed to equilibrate until the designated measurement temperature was reached. Temperature-dependent rheological behavior was first evaluated by temperature-sweep measurements from 25 to 37 °C at a heating rate of 0.5 °C/min, with a constant frequency of 1 Hz and strain of 1%. Frequency-sweep measurements were subsequently performed at 37 °C over an angular frequency range of 0.1 to 100 rad/s at a constant strain of 1%, selected within the linear viscoelastic region, to determine the storage (*G*′) and loss (*G*″) moduli [22]. For time-sweep measurements, the sample was heated from 25 to 37 °C and subsequently maintained at 37 °C. The storage (*G*′) and loss (*G*″) moduli were continuously monitored at a constant frequency of 1 Hz and a strain of 1%. The apparent network-formation time was defined as the elapsed time from reaching 37 °C to the point at which *G*′ exceeded *G*″.

### Small-angle x-ray scattering

Samples for nanoscale structure analysis were scanned using quartz capillary (1.5 mm o.d.). Samples were measured using a custom-built small-angle x-ray scattering (SAXS) machine. The x-ray source (Xenocs, France) has an average photon energy of 8.05 keV with an estimated beam size (full width at half maximum) of 1 mm × 1 mm (H × V). 2D scattering images were radially averaged via Fit2D software upon acquisition on an Eiger 1M detector (Dectris, Switzerland).

### Cytocompatibility test

Human dermal fibroblasts (HDFs) (1.0 × 10^4^ cells/well) were seeded into 24-transwell plates and allowed to adhere for 24 h, followed by exposure to the PCL scaffold placed in the inserts for 24 h at 37 °C. Finally, cell viability was evaluated by Cell Counting Kit-8 (CCK-8; Dojindo) assay, and cytocompatibility was further assessed by Live/Dead staining (Thermo Fisher Scientific) [23].

### In vitro blood–polymer interaction assay

Fresh sheep whole blood (Kisan bio) [24] was used for the in vitro blood–PCL interaction studies because it allowed sufficient and reproducible blood volumes to be obtained for repeated assays under controlled conditions. Mouse blood was used for the in vivo experiments to maintain species compatibility with the murine model. Experiments were performed in 24-transwell plates using 2 experimental protocols: premixed and sequential. In both cases, PCL scaffolds and blood were placed in the upper inserts, while 1× Dulbecco’s phosphate-buffered saline (DPBS) was added to the bottom wells to collect released blood-derived components. In the premixed protocol, blood was mixed with the scaffold prior to incubation, whereas in the sequential protocol, blood was deposited stepwise onto the PCL scaffold surface. All samples were incubated under static conditions at 37 °C. Nanoscale structural organization was characterized by SAXS, whereas morphology and elemental composition were analyzed by SEM/EDX. Blood components released from the scaffold into the DPBS were quantified by measuring methemoglobin (Met-Hb) absorbance at 630 nm [25].

### In vivo bruise formation and resolution assessment

All animal experiments were conducted in accordance with the Animal Protection Act and the Laboratory Animal Act of the Republic of Korea. The experimental protocols were reviewed and approved by the Institutional Animal Care and Use Committee (IACUC) of KIST (KIST-IACUC-2025-027). Adult male C57BL/6 mice (9 weeks old, DBL, Chungbuk, Korea) were anesthetized, and the dorsal skin was shaved and disinfected prior to injection. Whole blood was collected from mice via retro-orbital bleeding under anesthesia and immediately used for subsequent experiments.

For subcutaneous administration, injections were performed at predetermined dorsal sites, with the total injection volume maintained consistently across all groups (Table 1). All injections were delivered as a bolus into the subcutaneous space using a 31G, 8-mm needle. A within-animal experimental design was used, in which each mouse received all 4 experimental conditions at separate dorsal injection sites. A total of 6 mice were used, with 3 mice assigned to each collection time point (day 5 and day 10). Thus, each experimental condition included 3 independent biological replicates at each time point (*n* = 3 per group per time point). Four experimental groups were established: (a) blood + saline as a vehicle control, (b) blood premixed with liquid-type PCL immediately prior to injection, (c) sequential injection of blood followed by PCL, and (d) sequential injection of PCL followed by blood. The sequential groups were designed to evaluate the effect of the order of blood–PCL contact at the same injection site. Injection sites were monitored for up to 10 days following administration. The sites were visually inspected and photographed immediately after injection (day 0) and at designated time points up to day 10. This within-animal design enabled direct comparison among the 4 experimental conditions while reducing variability associated with interanimal differences.

**Table 1.** Injection composition, total volume, and sequence for each experimental group in the in vivo bruise model

Group	Saline	Blood	PCL	Total injection volume	Injection sequence
Group 1	75 μl	75 μl	–	150 μl	–
Group 2	–	50 μl	100 μl	150 μl	–
Group 3	–	50 μl	100 μl	150 μl	Blood → PCL
Group 4	–	50 μl	100 μl	150 μl	PCL → Blood

For interaction analysis, tissues were collected on days 5 and 10 postinjection. Three independent animals were analyzed at each time point (*n* = 3 per experimental condition at each time point). Each mouse was considered one independent biological replicate for each experimental condition. Multiple tissue sections or microscopic fields obtained from the same animal were treated as technical measurements and were not considered independent biological replicates. Where multiple measurements were obtained from the same animal, they were averaged at the animal level prior to statistical analysis. To elucidate the microstructural and molecular features underlying blood–PCL scaffold interactions and the associated tissue responses, harvested tissues were analyzed using cryo-SEM, Raman spectroscopy, histological staining, immunofluorescence staining for CD68 (Santa Cruz Biotechnology, sc-20060, A594), CD86 (Invitrogen, 14-0862-82), and CD206 (Abcam, ab64693), and Perls’ Prussian blue staining using an Iron Stain Kit (Abcam, ab150674).

### Statistical analysis

Data are presented as mean ± SD. Statistical significance was assessed using one-way analysis of variance (ANOVA) or 2-sample *t* tests (OriginLab, USA), as appropriate. For comparisons among multiple experimental conditions, one-way ANOVA followed by Tukey’s multiple-comparison test was used where appropriate. For quantitative in vivo endpoints, measurements from multiple sections or fields were averaged within each animal and condition before analysis. Comparisons among the 4 conditions were performed using one-way repeated-measures ANOVA, with animal identity as the matching factor, followed by Tukey’s multiple-comparison test. Statistical analyses were performed using OriginLab (USA). Statistical significance was defined as *P < 0.05, **P < 0.01, and ***P < 0.001. Exact sample sizes and replicate definitions are provided in the corresponding figure legends.

## Results and Discussion

### Network formation of liquid-type PCL

Liquid-type PCL is formulated as an injectable solution in the sol state at ambient temperature, facilitating precise delivery into dermal layers with minimal trauma. Upon exposure to physiological temperature (37 °C), the formulation undergoes a rapid and controlled sol-to-network transition, yielding a mechanically robust hydrogel depot at the target site. The temperature-induced network formation is visually confirmed by the transition from a free-flowing sol at 25 °C to self-supporting scaffold at 37 °C (Fig. 2A). Cryo-SEM analysis of the in vitro-formed scaffold reveals a highly interconnected porous architecture with pore sizes ranging from approximately 1 to 5 μm and an average pore diameter of 2.54 ± 1.00 μm (Fig. S1). Such a porous network may facilitate water retention and provide structural spaces for the entrapment and retention of blood components within the scaffold matrix.

**Fig. 2.**
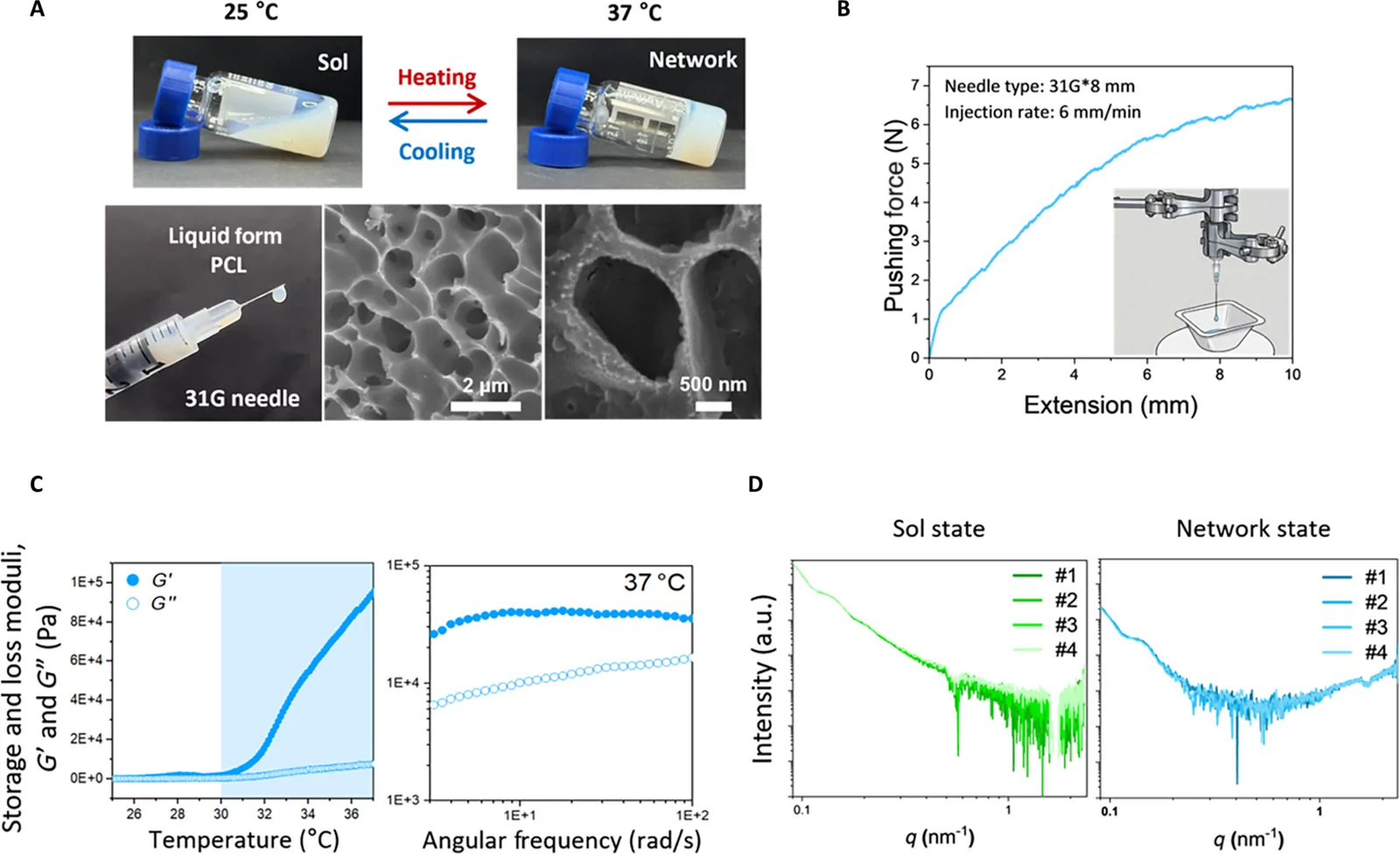
Thermoresponsive structural, rheological, and nanoscale characterization of the injectable PCL scaffold. (A) SEM images showing the porous microstructure of the in vitro formed PCL scaffold. Scale bars, 2 μm (left) and 500 nm (right). (B) Injection force–displacement curve recorded during extrusion of the liquid-type PCL formulation through a 31G needle at a crosshead speed of 6 mm/min. (C) Temperature-sweep rheological analysis showing the temperature-dependent changes in storage modulus (*G*′) and loss modulus (*G*″) of the liquid-type PCL formulation. A pronounced increase in *G*′ beginning at approximately 30 °C indicates the onset of temperature-induced network strengthening. The frequency sweep was performed at 37 °C. (D) Small-angle x-ray scattering (SAXS) profiles revealing nanoscale structural organization of the PCL network. SAXS measurements were performed using 4 independently prepared samples (*n* = 4).

Quantitative assessment of injection dynamics demonstrates the injectability of the liquid PCL formulation loaded in 1-ml syringes (Fig. 2B). Extrusion requires only modest forces (3 to 8 N over 10-mm displacement at 6 mm/min), supporting the feasibility of administration through a fine-gauge needle.

Temperature-sweep rheometry showed a pronounced increase in the storage modulus (*G*′) above approximately 30 °C, indicating temperature-induced network strengthening of the liquid-type PCL formulation (Fig. 2C). At 37 °C, *G*′ remained higher than *G*″ across the tested frequency range, consistent with the formation of an elastic network structure [26]. To further evaluate the kinetics of network formation under physiological temperature, time-sweep rheological measurements were performed during the temperature transition from 25 to 37 °C. A *G*′–*G*″ crossover was observed within 60 s after reaching 37 °C, indicating rapid development of elastic-dominant behavior under the tested in vitro conditions (Fig. S2). Compression testing revealed a compressive modulus of 26.3 kPa for the hydrogel, which is within the range reported for subcutaneous tissues (Fig. S3) [27]. This compliant mechanical property may facilitate conformity to the surrounding tissue and contribute to improved comfort following administration.

The SAXS measurements further demonstrate structural variations between the sol and hydrogel states. The sol state displays scattering profiles with weak and broad oscillations, which are attributed to the particle form factor. In contrast, the network phase is characterized by a pronounced low-*q* upturn, which reflects the formation of larger, spatially correlated network structures or aggregates that underpin the hydrogel’s mechanical integrity (Fig. 2D).

The capability of the liquid PCL formulation to transition into a porous scaffold upon exposure to physiological conditions supports its potential to form a localized scaffold after injection. The combined rheological, microstructural, and SAXS findings demonstrate temperature-dependent changes in the organization and mechanical behavior of the PCL formulation.

### In vitro biocompatibility and blood–polymer interaction tests

The biocompatibility test on HDFs confirmed high cell viability across all concentrations tested, consistent with PCL’s well-known favorable biocompatibility and low cytotoxicity profile (Fig. 3A) [28]. Specifically, indirect cytotoxicity assays employing scaffold extracts across a concentration gradient (0, 50, 100, 200, and 500 μl/ml) revealed consistently high cell viability exceeding 90% after 24 h exposure, as quantified by CCK-8 and normalized to untreated controls. These findings corroborate the well-established biocompatibility profile of PCL, a semicrystalline polyester renowned for its slow biodegradability, mechanical robustness, and minimal inflammatory response in soft tissue applications. Several medical devices containing PCL as an ingredient have been approved by the United States Food and Drug Administration [29]. These results support the safe use of the PCL polymer matrix in skin applications without inducing adverse cellular responses. Beyond cytocompatibility, however, the 3-dimensional network formed by the thermoresponsive PCL formulation may also alter the local transport and retention of blood components following vascular injury.

**Fig. 3.**
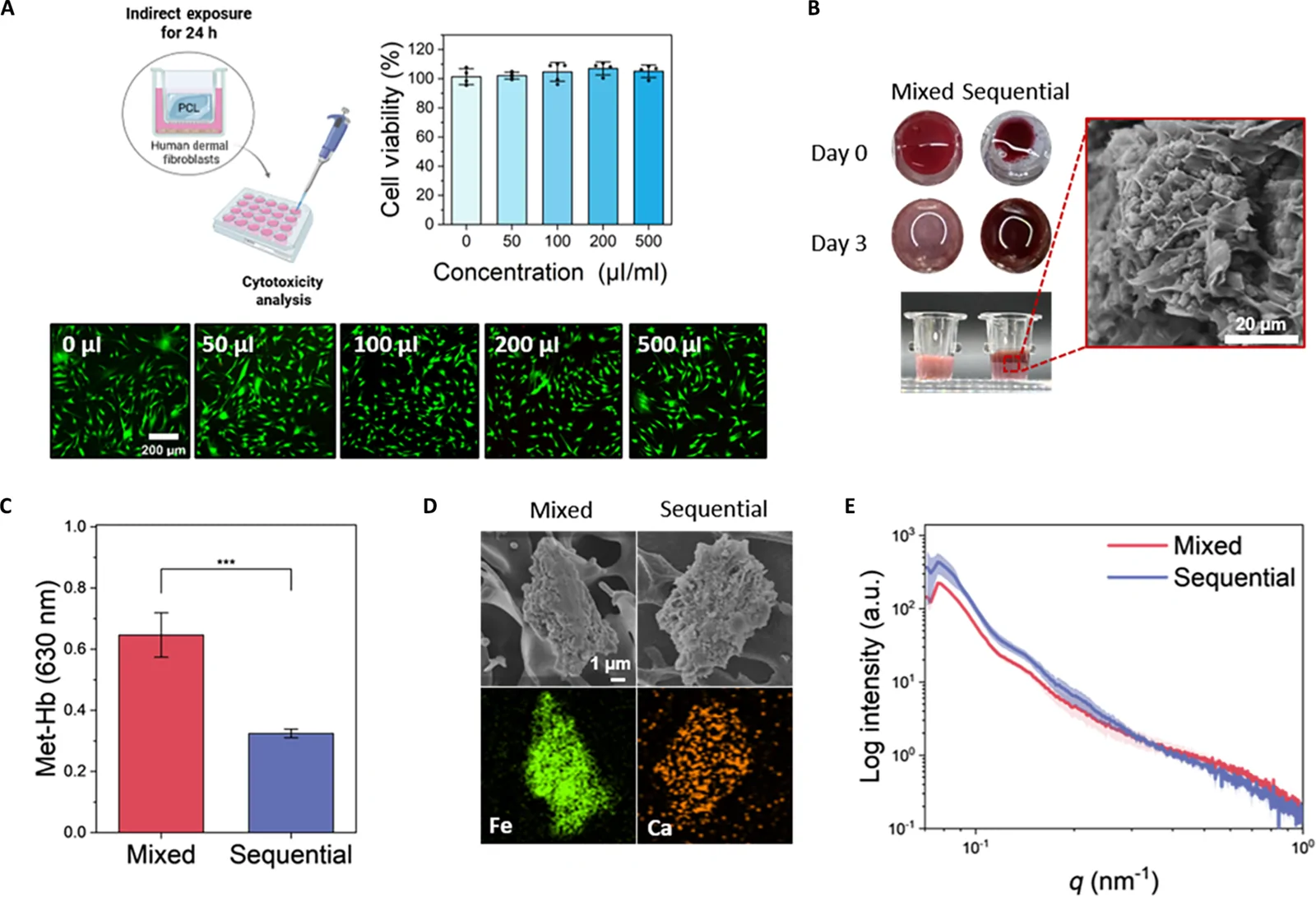
In vitro cytocompatibility and blood–PCL scaffold interactions with structural and molecular analyses. (A) Indirect cytotoxicity assessment of the PCL scaffold using human dermal fibroblasts after 24 h exposure, showing cell viability under different concentrations. CCK-8 assays were performed using 3 independent samples (*n* = 3) for each condition, and data are presented as mean ± SD. Representative Live/Dead fluorescence images are shown. Scale bar, 200 μm. (B) Representative in vitro digital images illustrating blood–PCL scaffold interactions, along with SEM images of blood components adsorbed onto the PCL scaffold surface. Scale bar, 20 μm. (C) Quantitative analysis of oxidized hemoglobin levels in the mixed and sequential blood–PCL scaffold interaction groups. Statistical comparisons were performed using two-sample *t* test. Experiments were performed using 3 independently prepared samples (*n* = 3). ***P < 0.001. (D) Representative SEM images and corresponding EDX elemental maps showing Fe in the mixed sample and Ca in the sequential sample. Additional elemental maps and quantitative compositions are provided in Fig. S7, where Ca in the mixed sample and Fe in the sequential sample are reported as not detected (ND). Scale bar, 1 μm. (E) SAXS profiles comparing nanoscale structural features of the mixed and sequential groups.

Polymer network architecture and density can influence the transport and local retention of incorporated components [30–32]. Based on this consideration, we next investigated whether the temporal sequence of blood contact relative to PCL scaffold formation influences blood–polymer interactions. To elucidate the mechanistic basis of blood–polymer interactions pertinent to inadvertent vascular puncture during injection—a common clinical occurrence in aesthetic dermatology—2 paradigmatic experimental setups were systematically investigated: (a) a “mixed” condition where liquid-type PCL was admixed with whole blood at room temperature prior to temperature-induced network formation, and (b) a “sequential” condition involving introduction of blood into a preformed PCL hydrogel scaffold. Both constructs were incubated for 3 days under physiological conditions (37 °C, 5% CO_2_, humidified atmosphere) to recapitulate postinjection environment. Gross morphological examination via digital microscopy of the blood–PCL constructs after 3 days revealed distinct color evolution dynamics reflective of hemoglobin stability (Fig. 3B). The mixed condition exhibited accelerated decolorization with pronounced leaching of red hue, suggesting greater loss or transformation of blood-derived pigments, whereas the sequential condition showed better preservation of red coloration with less visible fading, consistent with prolonged retention of blood-associated components within the preformed scaffold. Complementary SEM imaging of the sequential condition further revealed blood-derived aggregates and cellular structures morphologically resembling erythrocytes, together with fibrin-like deposits on the scaffold surfaces. To further examine whether the distinct blood–scaffold interactions were accompanied by differences in hemoglobin stability, we next evaluated hemoglobin oxidation and release over time.

Hemoglobin oxidation was assessed spectrophotometrically by measuring absorbance at 630 nm (*A*_630_), a characteristic wavelength associated with Met-Hb accumulation resulting from oxidation of ferrous (Fe^2+^) to ferric (Fe^3+^) heme [25,33]. *A*_630_ values were markedly elevated in the mixed group compared to the sequential counterpart (Fig. 3C and Fig. S4), indicating greater hemoglobin oxidation in the mixed condition. Figure S4A presents the temporal evolution of the *A*_630_/*A*_576_ ratio, used as a relative indicator of hemoglobin oxidation over time. The mixed group exhibited a high *A*_630_/*A*_576_ ratio from the earliest time point, consistent with rapid hemoglobin oxidation, whereas the sequential group showed a lower ratio at 6 and 12 h followed by a gradual increase after 24 h, indicating a delayed oxidation profile. Figure S4B shows the cumulative release of total hemoglobin (oxyhemoglobin + Met-Hb) over time. The mixed group released more hemoglobin throughout the observation period, whereas the sequential group showed consistently lower release, consistent with greater hemoglobin retention within the scaffold-associated environment. Collectively, these findings suggest that the preformed scaffold in the sequential condition may favor the retention of blood-derived components and delay their oxidation and release. Supporting rheological measurements showed that blood-mixed PCL exhibited a lower storage modulus at 37 °C than PCL alone (Fig. S5). The sequential blood–PCL constructs also retained more mass during prolonged incubation than the mixed constructs (Fig. S6A). Because wet mass was measured, the observed changes reflect the combined effects of fluid uptake or loss and changes in construct constituents. In a separate experiment, blood-free PCL scaffold degradation showed only limited changes over the investigated period (Fig. S6B), suggesting that polymer degradation alone could not fully explain the difference in construct mass. Together with the greater cumulative hemoglobin release from the mixed constructs (Fig. S4B), these findings suggest that the release of blood-derived components contributed to the observed mass changes, although changes in water content and the loss of other soluble constituents may also have contributed. These complementary observations support differences in blood-component retention and release between the mixed and sequential conditions.

Cryo-SEM/EDX analysis revealed differences in the local elemental composition of the analyzed blood–PCL regions (Fig. 3D and Fig. S7). In the analyzed regions, Fe was detected in the mixed sample (17.20 wt%) but was not detected in the sequential sample, whereas Ca was detected only in the sequential sample (0.47 wt%). These observations are consistent with differences in the local distribution of elements within the blood–PCL constructs, potentially reflecting differences in component organization associated with the sequence of blood–polymer contact. The compositions were normalized to the selected elements (C, O, Fe, and Ca). Because EDX probes localized regions, these findings do not establish differences in total blood retention or hemoglobin oxidation, and Fe nondetection does not imply its absence throughout the sequential construct.

At the nanoscale, SAXS measurements were performed to assess structural organization within the blood–PCL constructs (Fig. 3E). We hypothesized that the scaffold functions through a hierarchical architecture, where the nanostructural integrity within the pore walls—alongside microporosity—plays a pivotal role in mechanically stabilizing the sequestered blood components. Both the sequential and mixed constructs manifested characteristic periodic scattering motifs, indicating that the lamellar organization of the semicrystalline PCL domains was retained after blood incorporation. Notably, the sequential samples displayed substantially elevated scattering intensities across probed *q* ranges compared to the mixed samples. This difference is consistent with a larger volume fraction of ordered lamellar domains in the preformed scaffold, although absolute intensity comparisons are also sensitive to differences in sample thickness and composition. Specifically, in the sequential approach, blood infiltrates an already solidified PCL lattice with limited disruption of the preexisting lamellar organization. In contrast, the reduced scattering intensity in the mixed condition suggests that contemporaneous mixing of blood interferes with the polymer chain packing, possibly by limiting the development of ordered crystalline domains during network formation.

Collectively, these in vitro findings suggest that the timing of blood–polymer contact influences scaffold formation and stability, as well as the subsequent retention, oxidation, and release of blood-derived components. In particular, the preformed scaffold in the sequential condition was associated with greater retention and delayed oxidation and release of hemoglobin, supporting scaffold-mediated confinement as a potential contributor to these differences. These findings suggest that the sequence of blood and polymer contact may influence the evolution of bruising following skin booster injection.

Although bruise formation and resolution in vivo involve multiple physiological processes, the present in vitro system was intentionally simplified to isolate and compare blood–polymer interactions under controlled conditions. To determine whether the interaction-dependent differences observed in vitro were also evident under physiologically relevant conditions, complementary in vivo experiments were performed using a murine subcutaneous injection model.

### In vivo evaluation of bruise formation and resolution

To systematically investigate how the temporal sequence of blood–polymer contact affects bruise formation and local tissue responses, we employed a controlled murine dorsal subcutaneous injection model and monitored the injection sites over a 10-day period. This model was not intended to reproduce vascular injury itself, which occurs unpredictably during clinical injection, but rather to isolate the downstream interaction between extravasated blood and the injectable polymer under controlled conditions. In clinical practice, vascular disruption may occur before, during, or shortly after material deposition, resulting in distinct temporal patterns of blood–polymer contact. Accordingly, the present model was designed to model these distinct interaction sequences while minimizing interanimal variability. The 10-day observation period was intended to compare relative differences in bruise resolution among the experimental conditions and was not designed to reproduce clinically persistent bruising lasting for several weeks or longer.

To represent these clinically relevant blood–polymer interaction scenarios, the mixed, Blood → PCL, and PCL → Blood groups were designed as complementary variants of the timing of blood exposure relative to product delivery. The mixed condition models a scenario in which vascular injury occurs essentially synchronously with product injection, such that blood interpenetrates the polymer while it remains in the liquid state, prior to network formation. The Blood → PCL condition represents intraprocedural bleeding, in which extravasated blood is present during scaffold formation, whereas the PCL → Blood condition represents postinjection bleeding, in which blood is introduced after the scaffold has already formed, as may occur during a subsequent needle pass at the same site. By comparing these conditions within the same animal, the model enables the effect of blood–polymer contact sequence to be evaluated while reducing variability arising from differences among individual animals. The administered blood volume was selected to provide a reproducible and measurable bruise response under controlled conditions and was not intended to quantitatively reproduce the amount of vascular leakage occurring clinically. Therefore, this model should be regarded as a controlled platform for investigating the temporal sequence of blood–polymer interactions rather than as a direct reproduction of clinical bruising. Thus, 4 experimental groups were established: (a) blood + saline (vehicle control), (b) blood mixed with liquid PCL, (c) sequential injection of blood followed by liquid PCL, and (d) sequential injection of liquid PCL followed by blood (Fig. 4A).

**Fig. 4.**
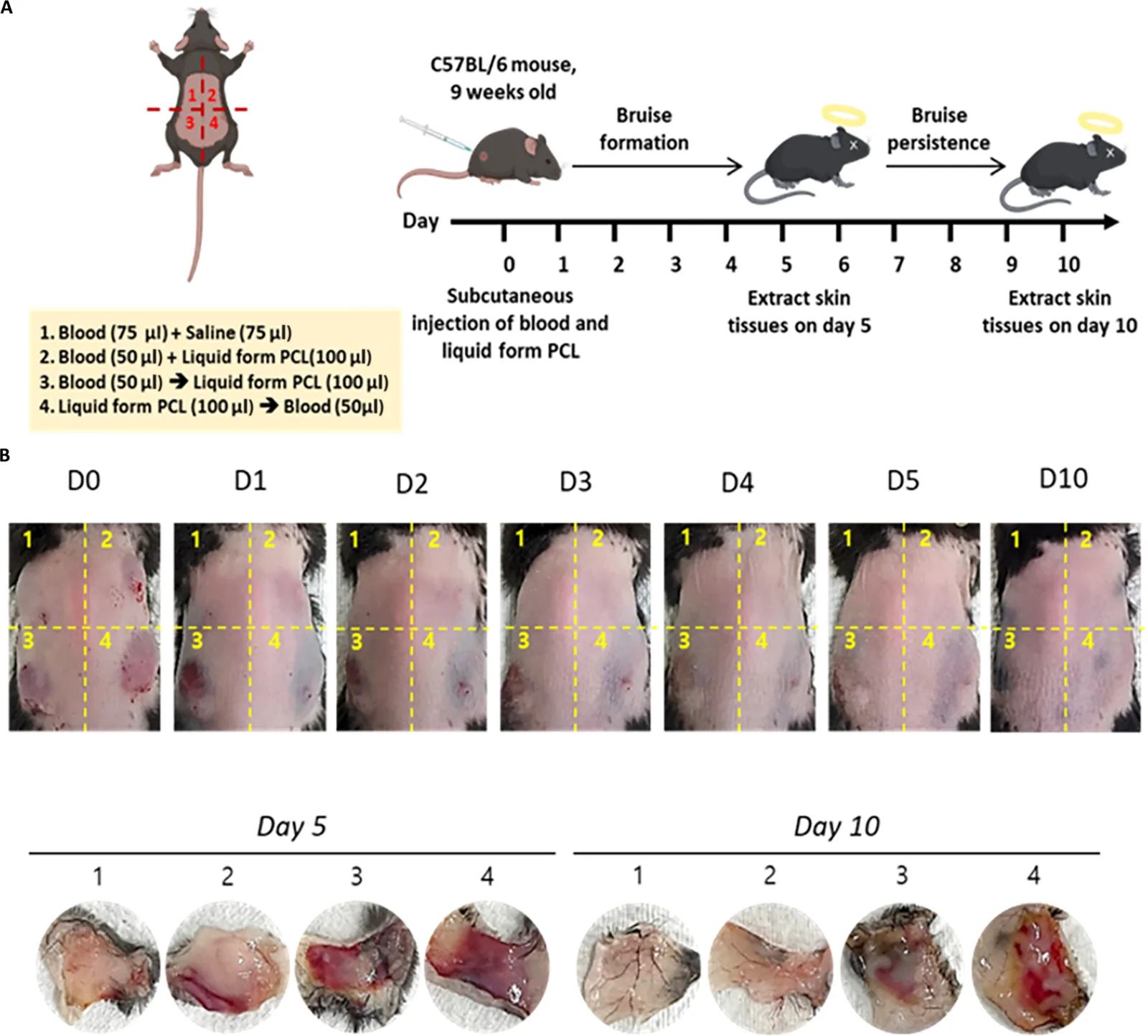
In vivo experimental design and longitudinal evaluation of bruise formation and resolution. (A) Schematic illustration of the in vivo bruise model and experimental timeline. Four different injection protocols were evaluated: (1) blood mixed with saline, (2) blood mixed with liquid-form PCL, (3) sequential injection of blood followed by liquid-form PCL, and (4) sequential injection of liquid-form PCL followed by blood. Injections were performed at defined dorsal sites to enable intra-animal comparison. (B) Representative gross photographs of the dorsal skin showing bruise formation and progression up to day 10 postinjection, together with representative images of harvested tissues collected on days 5 and 10. Four different injection conditions (groups 1 to 4) were administered to separate dorsal sites within each animal. Three independent animals were analyzed at each time point (*n* = 3 per group per time point).

The total injection volume was maintained at 150 μl across all groups, although the vehicle control contained a greater absolute volume of blood than the PCL-containing groups. Bruise resolution was evaluated as the temporal change relative to the initial postinjection condition, thereby reducing the influence of differences in the initial blood load. Macroscopic observations revealed clear differences between the mixed protocol (group 2) and the sequential injection protocols (groups 3 and 4) (Fig. 4B). The mixed group exhibited minimal and localized bruising that resolved rapidly, showing a tissue appearance comparable to the vehicle control (group 1) by day 5. In contrast, the sequential groups developed markedly more pronounced and persistent bruising, with hemorrhage remaining visible through day 10. Consistent with these observations, an ImageJ-based color analysis demonstrated sustained redness intensity in the sequential groups, particularly in group 3, whereas the blood-only and mixed groups showed a more rapid decline over time (Fig. S8). Notably, despite receiving a greater absolute volume of blood, the vehicle control exhibited more rapid bruise resolution than the PCL-containing sequential groups, suggesting that the initial blood volume alone is unlikely to account for the observed differences in bruise persistence.

### In vivo blood–polymer interaction and heme degradation

The underlying mechanisms driving these macroscopic differences were further elucidated through microstructural and molecular analysis using cryo-SEM and Raman spectroscopy. Cryo-SEM imaging of tissue harvested 24 h after in vivo injections without blood demonstrated a well-formed porous PCL scaffold, closely resembling the pore structure observed in the in vitro cryo-SEM images (Fig. 5A). These observations confirm that a porous PCL scaffold forms under in vivo conditions within 24 h after injection.

**Fig. 5.**
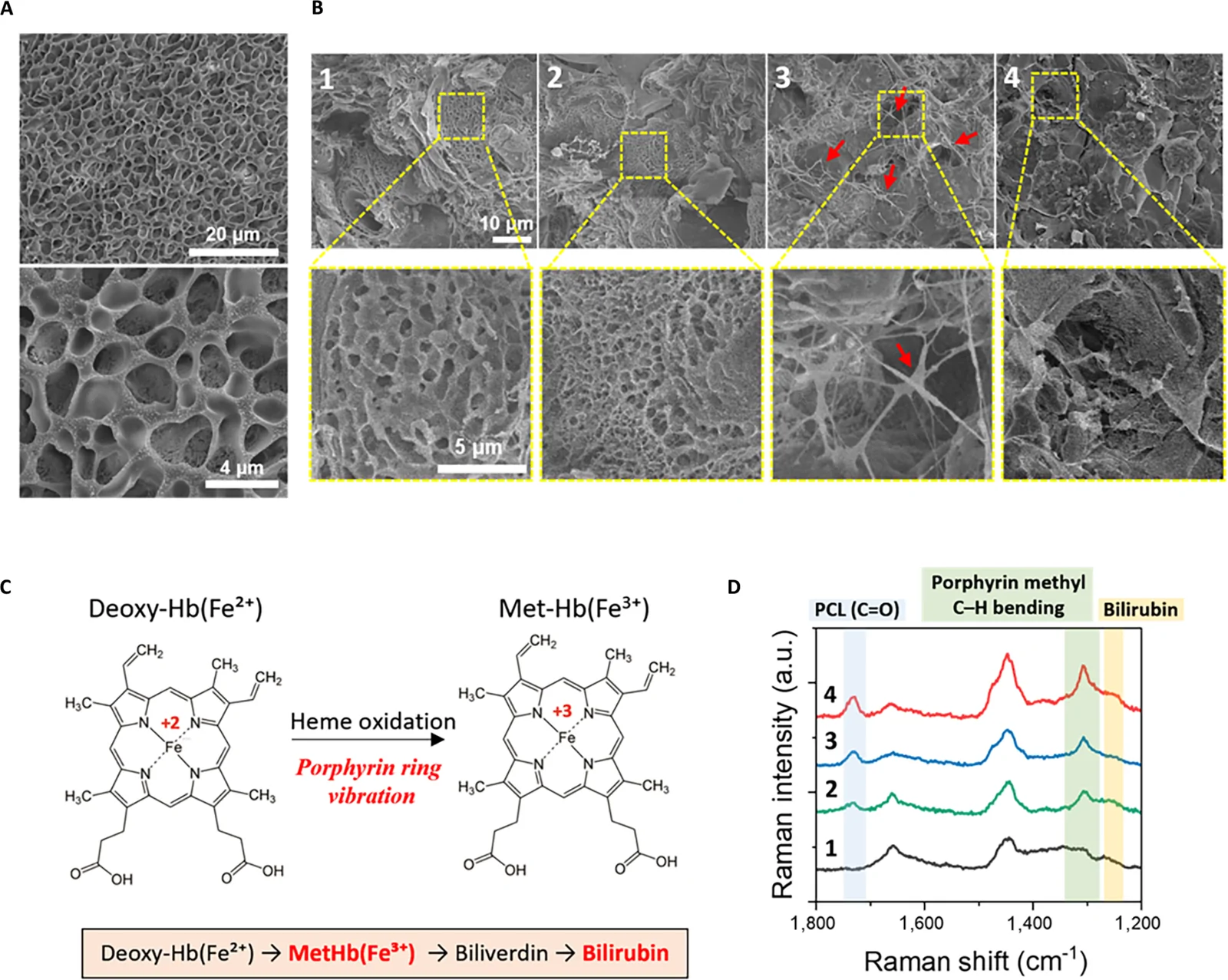
In vivo blood–PCL scaffold interactions and heme degradation analyzed by cryo-SEM and Raman spectroscopy. (A) Cryo-SEM images of the PCL scaffold retrieved in vivo 24 h after subcutaneous injection, showing the resulting scaffold microstructure after subcutaneous injection. Scale bars, 20 μm (top image) and 4 μm (bottom, higher-magnification image). (B) Cryo-SEM images of in vivo-treated tissues from different experimental groups 10 days postinjection, revealing distinct tissue–blood–scaffold interaction morphologies. Yellow dashed boxes indicate regions shown at higher magnification in the bottom panels. Red arrows indicate fibrin-like filamentous structures associated with retained blood-derived components within the PCL-containing tissue. Scale bars, 10 μm (top) and 5 μm (bottom, higher-magnification images). (C) Schematic illustration of hemoglobin degradation and oxidation, depicting the conversion of Deoxy-hemoglobin (Fe^2+^) to Met-hemoglobin (Fe^3+^), accompanied by porphyrin ring vibration and subsequent formation of biliverdin and bilirubin. (D) Raman spectra of in vivo tissue samples highlighting characteristic peaks, indicating molecular changes associated with blood degeneration.

Subsequently, scaffold formation was evaluated in the presence of blood in our animal experiment groups 10 days postinjection, providing microstructural insight. Group 1 (blood + saline) exhibited tissue morphology similar to normal subcutaneous tissue, with no visible scaffold, indicating rapid blood clearance (Fig. 5B). In group 2, polymer scaffold formation was markedly impaired or absent, consistent with the inability of polymer chains to organize into a stable in vivo network when mixed with blood prior to scaffold formation. In contrast, groups 3 and 4, which received sequential injections, displayed distinct porous scaffold structures within the subcutaneous tissue. At higher magnification, filamentous structures morphologically resembling fibrin networks were observed in association with the scaffold matrix, most prominently in group 3 (red arrows in Fig. 5B), together with adherent blood-derived material. Because fibrin or fibrinogen staining was not performed, these features are described on a morphological basis only and are not taken as direct evidence of fibrin deposition. Their localization within and adjacent to the PCL network is nonetheless consistent with localized retention of blood-derived components in association with the formed scaffold. Together, these observations suggest that the timing and sequence of blood exposure influence scaffold architecture and are associated with prolonged retention of blood-derived components in vivo under sequential blood–polymer exposure conditions.

Hemoglobin contains a heme group composed of an iron ion coordinated within a porphyrin macrocycle. During hemoglobin degradation, the porphyrin ring structure is progressively broken down through enzymatic conversion to biliverdin and subsequently bilirubin. Unlike hemoglobin-derived heme, bilirubin no longer retains the intact porphyrin macrocycle. Therefore, persistence of porphyrin-associated Raman features may be consistent with the presence of heme-containing blood components (Fig. 5C) [34]. Raman spectra revealed characteristic signals corresponding to both the PCL scaffold and porphyrin-containing blood components (Fig. 5D). The peak observed at approximately 1,730 cm^−1^ was attributed to the ester carbonyl (C=O) stretching vibration of PCL, confirming the presence of the scaffold material. Porphyrin-associated signals were lower in groups 1 and 2 than in groups 3 and 4 [35,36]. Given that hemoglobin contains an intact heme porphyrin structure, whereas this structure is progressively lost during blood degradation and clearance, the diminished hemoglobin-associated signal in groups 1 and 2 suggests blood removal from the injection site. Conversely, the stronger hemoglobin-related signals observed in groups 3 and 4 indicate delayed clearance of blood components within the scaffold microenvironment.

### Histological evaluation of tissue response and macrophage recruitment

The histological evaluation further supported this mechanism of delayed clearance (Fig. 6A). In the sequential groups, substantial macrophage cell infiltration was observed alongside intact or slowly degrading red blood cell (RBC) aggregates that remained localized within the subcutaneous layer even on day 10. The dense inflammatory cell infiltration in these groups is likely a response to the sustained presence of these sequestered blood components, which the body cannot rapidly clear due to the physical entrapment within the preformed PCL scaffold. In contrast, the mixed group showed accelerated resolution of the bruise. Quantitative analysis of hematoxylin and eosin-stained sections revealed significantly higher RBC retention in groups 3 and 4, whereas only minimal RBC-positive areas were observed in groups 1 and 2. Together, these findings suggest that differences in scaffold organization associated with the timing of blood–polymer contact may influence the local retention and subsequent clearance of erythrocytes.

**Fig. 6.**
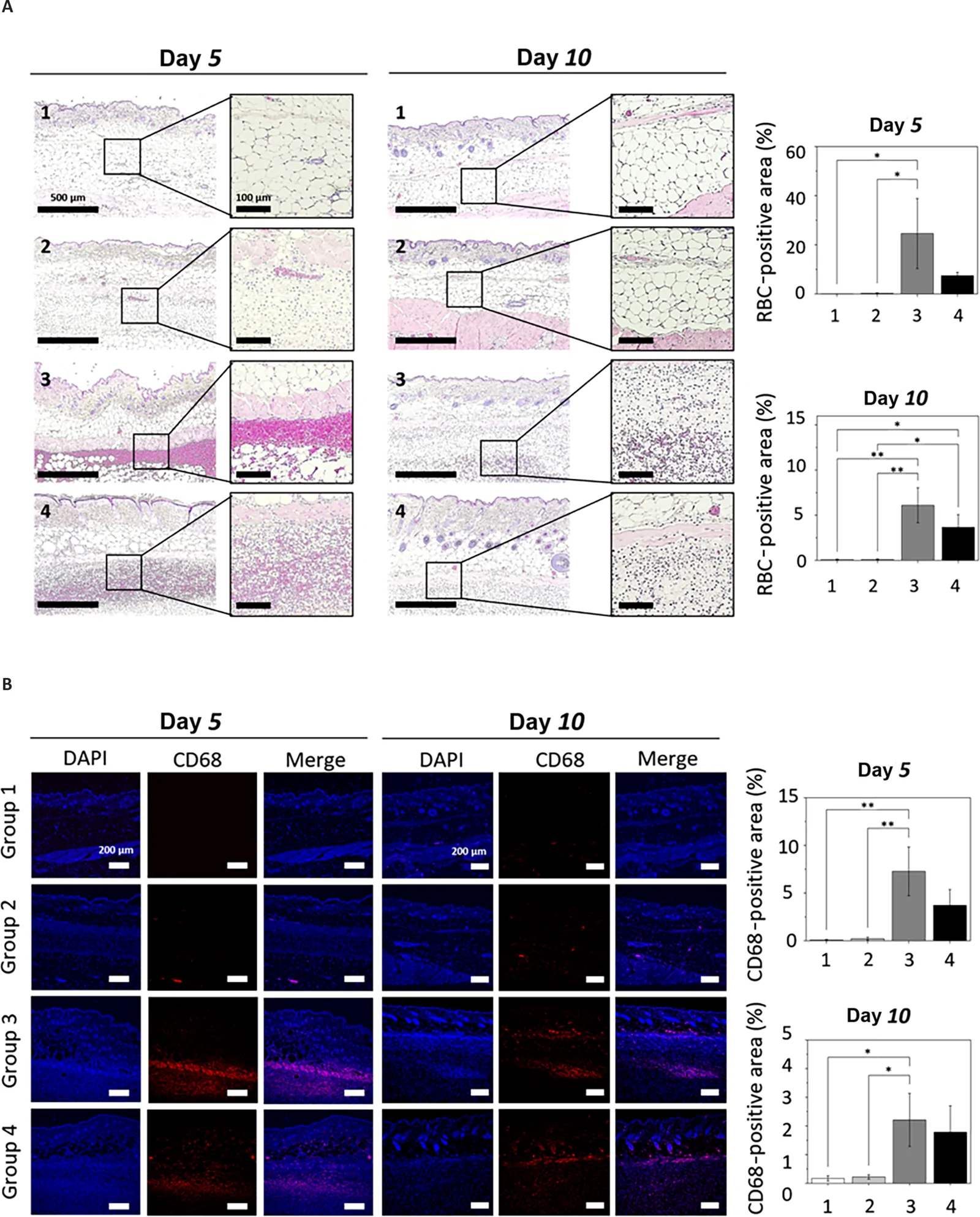
Histological evaluation of skin tissues at different time points following in vivo bruise induction. (A) Representative hematoxylin and eosin (H&E)-stained images of skin tissues harvested on days 5 and 10 from each experimental group, together with semiquantitative analysis of RBC-positive areas. Low-magnification images show the overall tissue architecture, while boxed regions indicate magnified regions highlighting hemorrhage-related features. Scale bars, 500 μm (low-magnification images) and 100 μm (higher-magnification images). Quantitative data were obtained from 3 biological replicates per group (*n* = 3) and are presented as mean ± SD. Statistical comparisons were performed separately at each time point using one-way repeated-measures ANOVA followed by Tukey’s multiple-comparison test. *P < 0.05 and **P < 0.01. (B) Representative CD68 immunofluorescence images and quantification of CD68-positive area. Nuclei were counterstained with DAPI. Groups 3 and 4 showed stronger CD68-positive signals than groups 1 and 2, suggesting increased macrophage recruitment within the scaffold-associated tissue environment. Scale bars, 200 μm. Quantitative data were obtained from 3 biological replicates per group (*n* = 3) and are presented as mean ± SD. Statistical comparisons were performed separately at each time point using one-way repeated-measures ANOVA followed by Tukey’s multiple-comparison test. *P < 0.05 and **P < 0.01.

CD68 immunofluorescence staining revealed minimal macrophage infiltration in groups 1 and 2, whereas groups 3 and 4 exhibited markedly stronger CD68-positive signals within the subcutaneous tissue (Fig. 6B). These findings suggest sustained macrophage recruitment associated with retained blood-derived components and delayed hemoglobin clearance within the scaffold microenvironment. The persistent macrophage presence is consistent with delayed bruise resolution and delayed hemoglobin degradation observed in the sequential injection groups. To further characterize the local inflammatory response, additional immunofluorescence staining for CD86 and CD206 was performed on days 5 and 10 [37]. The CD206-positive/CD86-positive area ratio was lower in both sequential groups on day 5, whereas higher ratios were observed on day 10, indicating time- and treatment-dependent changes in the balance of inflammatory- and repair-associated marker expression (Fig. S9). These findings suggest an evolving macrophage-associated response during delayed blood clearance [38–40], although CD86/CD206 staining alone does not establish macrophage-specific polarization. Perls’ Prussian blue staining further revealed greater Prussian blue-positive iron deposits in the sequential groups on day 10, whereas staining was minimal in the control and mixed groups (Fig. S10). This pattern is consistent with the persistence of iron/hemosiderin-containing degradation products and delayed local processing and clearance of retained blood components.

Both sequential injection groups exhibited delayed RBC clearance and delayed bruise resolution compared with the mixed condition; however, group 3 consistently showed greater RBC retention and more persistent discoloration than group 4. These findings suggest that the timing of blood exposure relative to scaffold formation influences the extent of blood sequestration within the PCL network. In group 3, erythrocytes were present during the scaffold-forming process and may therefore have become incorporated into the developing PCL network. Such incorporation could facilitate more effective entrapment of blood components within the scaffold architecture and delay their subsequent clearance. In contrast, in group 4, erythrocytes were introduced after scaffold formation and therefore had to enter an already established pore network. Under these conditions, some blood components may be retained within the scaffold, whereas others may remain distributed within the surrounding tissue, resulting in less effective sequestration. Consistent with this interpretation, group 3 exhibited greater RBC retention and more delayed bruise resolution than group 4, suggesting that blood incorporation during scaffold formation may be an important determinant of scaffold-mediated blood retention.

Despite these differences in administration sequence between groups 3 and 4, both sequential injection conditions exhibited substantially greater RBC retention and more delayed bruise resolution than the mixed condition. In contrast, the mixed condition was associated with less organized scaffold formation and lower retention of blood-derived components, which may contribute to their more rapid clearance.

The in vitro findings further revealed distinct hemoglobin oxidation behaviors between the mixed and sequential conditions. In the mixed group, accelerated Met-Hb generation and more rapid spectral transitions were observed, a pattern that paralleled the relatively faster bruise resolution observed in vivo. In contrast, the sequential groups exhibited greater retention of blood-derived components together with delayed changes in hemoglobin oxidation and release, indicating delayed clearance of blood-derived components within the scaffold-associated microenvironment.

Consistent with these observations, the sequential injection groups in vivo demonstrated delayed discoloration resolution, increased RBC retention, and sustained accumulation of CD68-positive macrophages within the tissue, together with time-dependent changes in CD86/CD206 expression and persistent Prussian blue-positive iron deposits. Although the in vitro experiments were conducted under simplified static conditions, whereas the in vivo environment involves dynamic perfusion, immune responses, and complex tissue interactions, the overall trends observed across both systems consistently support delayed processing and clearance of scaffold-associated blood components involving both physical retention and inflammatory/remodeling responses.

Although the present findings support scaffold-mediated physical retention of blood components as an important contributor to delayed clearance, this process is unlikely to occur independently of physiological hemostatic responses. Contact between extravasated blood and the scaffold may also promote coagulation and fibrin network formation, which could further stabilize erythrocytes and other blood-derived components within the local matrix. Consistent with this possibility, dense, clot-like regions were observed within the PCL scaffold in the sequential injection groups. However, because coagulation activation and fibrin deposition were not directly quantified in the present study, their relative contribution cannot be distinguished from that of physical scaffold-mediated confinement. Therefore, coagulation/fibrin-mediated retention should be considered a potential co-contributing mechanism rather than a mechanism directly demonstrated by the current data. Future studies incorporating fibrin or fibrinogen staining and quantitative assessment of coagulation-related markers will be needed to further resolve their respective contributions.

From a clinical perspective, these findings provide experimental support for the previously proposed hypothesis regarding delayed bruise resolution [17]. Prophylactic measures to prevent delayed bruise clearance following skin booster injections should focus primarily on minimizing vascular trauma and optimizing injection techniques. A preprocedural assessment of bleeding risk and medication history may be considered, with any modification of anticoagulant or antiplatelet therapy determined by the prescribing physician on an individual basis.

Delayed bruise-like discoloration is expected to undergo progressive resolution; however, the present study does not establish its long-term course or a direct temporal relationship with PCL scaffold degradation. However, since persistent bruising can lead to patient discomfort, preventive and therapeutic strategies should be considered to optimize clinical outcomes. The application of cold compresses and the use of vasoconstrictors, such as epinephrine combined with lidocaine, are recommended to reduce immediate bleeding risk [41]. During the procedure, the use of blunt-tipped, moderate-gauge cannulas and a slow, gentle retrograde injection technique is critical to avoid piercing vessels [41]. Furthermore, fanning techniques should be avoided in favor of smooth linear injections, and the material should ideally be placed in deeper layers, such as the subcutaneous plane, particularly in sensitive areas like the infraorbital region [17]. Research indicates that diluting the PCL formulation to create a less compact scaffold may also markedly reduce the likelihood of delayed blood clearance and persistent pigment entrapment.

As outlined in Byeon et al. [17], therapeutic management of persistent discoloration associated with delayed blood clearance is based on 4 primary mechanistic strategies designed to facilitate the clearance of trapped blood components from the PCL scaffold: pigment fragmentation, thermal loosening of the scaffold structure, mechanical dispersion, and chemical degradation. Reported approaches have included picosecond and Q-switched Nd:YAG lasers, sometimes in combination with other modalities. However, available evidence remains limited, and further clinical studies are required to establish comparative efficacy.

## Conclusion

This study elucidates critical factors influencing bruise formation and persistence following PCL-based skin booster injections. The temperature-responsive sol-to-network transition of liquid-type PCL enables facile injection and subsequent stable scaffold formation in vivo. Importantly, the interaction between the polymer network and extravasated blood components is strongly dependent on the sequence of administration. Coinjection of blood and PCL in the sol state interferes with optimal hydrogel network assembly, resulting in reduced erythrocyte retention and relatively faster bruise resolution. In contrast, sequential injection—either blood followed by PCL or PCL followed by blood—creates a microstructural environment that promotes retention of extravasated erythrocytes and delays their clearance, thereby delaying bruise resolution.

A limitation of the present study is that the in vivo observations were restricted to a 10-day period and therefore primarily reflect delayed bruise resolution rather than clinically persistent bruising. Although the sequential injection groups demonstrated sustained discoloration, increased RBC retention, and delayed hemoglobin processing within this timeframe, it remains unclear whether these findings translate into delayed discoloration over clinically relevant time scales. Importantly, persistence of the PCL scaffold does not necessarily imply permanent retention of blood-derived components. The decrease in RBC-positive areas from day 5 to day 10, together with Prussian blue staining, hemoglobin processing, and CD68/CD86/CD206 analyses, suggests ongoing processing of blood-derived components and macrophage-associated remodeling despite delayed clearance. Nevertheless, residual blood-derived products may contribute to prolonged discoloration, iron/hemosiderin accumulation, or sustained local inflammatory responses, warranting longer-term investigation. Another limitation concerns the kinetics of PCL network formation in vivo. Time-sweep rheological measurements showed a *G*′–*G*″ crossover within 60 s after reaching 37 °C, indicating rapid temperature-induced network formation in vitro. However, although a well-developed PCL scaffold was confirmed in vivo at 24 h after injection, the precise kinetics of network formation under in vivo conditions remain to be determined. Future studies with extended observation periods and clinically relevant models, together with direct assessment of oxygen and mass transport within the scaffold microenvironment, are needed to clarify both the long-term fate of retained blood-derived components and the mechanisms underlying delayed blood clearance and bruise resolution.

Collectively, these mechanistic insights highlight the importance of careful injection protocols to avoid unintended vascular injury and subsequent blood–polymer complexation that may exacerbate adverse outcomes. Our findings suggest that scaffold-mediated RBC retention may contribute to delayed blood clearance. Future studies should therefore investigate formulation strategies that generate a more open scaffold architecture, such as reducing polymer concentration or modifying network density, to facilitate the diffusion and clearance of extravasated blood. In addition, recent clinical observations have suggested that delayed bruising is less frequently observed when the material is distributed more diffusely rather than forming a dense localized scaffold. Therefore, alternative delivery approaches that promote broader material dispersion may further reduce localized blood entrapment. Furthermore, because postprocedural bruising is initiated by inadvertent vascular injury, optimization of injection techniques, including careful cannula-based delivery, slow injection rates, and avoidance of highly vascular regions, may help minimize blood–scaffold interactions and reduce the risk of delayed discoloration. Ultimately, an improved understanding of blood–polymer interactions will guide the rational design and clinical use of next-generation skin boosters, enhancing patient safety and therapeutic performance in aesthetic medicine.

## Data Availability

The raw data supporting the findings of this study are available from the corresponding authors upon reasonable request.
