# Supplementary material for: Mechanistic Insights into Delayed Bruise Clearance after Skin Booster Injection: Role of Blood–Polymer Complexation
Source: Biomater Res. 2026 Nov 12;30:0445. doi: 10.34133/bmr.0445 (PMC13756775; doi:10.34133/bmr.0445)
Supplement: Supplementary file 1 [file bmr.0445.f1.zip › old/Supporting Information_revision.docx]

SUPPLEMENTARY MATERIALS

**Mechanistic insights into delayed bruise clearance after skin booster injection: role of blood–polymer complexation**

*Soonjong Roh† ^1^, So-Jin Park† ^1, 2^, Seongryeol Ye ^1, 3^, Youngmee Jung ^1, 4, 10^, Chanjong Lee ^5, 6^, Olha Shestakova ^5^, Bilal J. Malik ^7^, Jimyung Seo* ^8^, Hojun Kim* ^9, 10^ and Jin Yoo* ^1, 10^*

¹ Center for Biomaterials, Korea Institute of Science and Technology (KIST), Seoul 02792, Republic of Korea.

² Department of Chemical Engineering, Chung-Ang University, Seoul 06974, Republic of Korea.

³ Department of Applied Bioengineering, Graduate School of Convergence Science and Technology, Seoul National University, Seoul 08826, Republic of Korea.

⁴ School of Electrical and Electronic Engineering, YU-KIST Institute, Yonsei University, Seoul 03722, Republic of Korea.

⁵ Dexlevo Inc., Medical Research Department, Seoul 06093, Republic of Korea.

⁶ 4-ever Clinic, Seoul 06049, Republic of Korea.

⁷ Sculpted by Doctors Clinic, Bolton, United Kingdom.

⁸ Galleria Dermatology Clinic, Seoul 06325, Republic of Korea.

⁹ Center for Advanced Biomolecular Recognition, Korea Institute of Science and Technology (KIST), Seoul 02792, Republic of Korea.

¹⁰ Division of Bio-Medical Science & Technology, KIST School, University of Science and Technology, Seoul 02792, Republic of Korea.

Address correspondence to: Jimyung Seo; seoj@kaist.ac.kr, Hojun Kim; cuboplex@kist.re.kr, and Jin Yoo; [jyoo@kist.re.kr](mailto:jyoo@kist.re.kr)

† These authors contributed equally to this work.

**1. Supplementary Figures**

**Fig. S1. Structural characterization of the liquid-type PCL scaffold.** Quantitative pore-size analysis of the in vitro-formed PCL scaffold based on cryo-SEM images. Data are presented as mean ± SD (n = 3).

**Fig. S2**. **Time-sweep rheological analysis of the PCL formulation at 37 °C.** The storage modulus (G’) and loss modulus (G”) were monitored as a function of time after exposure to physiological temperature. A G’-G” crossover was observed within 60 s, indicating rapid temperature-induced network formation under in vitro conditions. The arrow indicates the approximate crossover point.

**Fig. S3**. **Representative compressive stress–strain curve of the liquid-form PCL scaffold.** The scaffold exhibited a compressive modulus of 26.3 kPa, indicating soft mechanical properties comparable to those of subcutaneous soft tissue.

**Fig. S4. Hemoglobin oxidation and clearance profiles in mixed and sequential blood–PCL interaction conditions.** (A) Temporal changes in the ratio of methemoglobin relative to total hemoglobin, calculated from the absorbance signals of oxyhemoglobin (576 nm) and methemoglobin (630 nm). The increase in methemoglobin reflects the relative degree of hemoglobin oxidation within the scaffold-associated environment. (B) Cumulative release of total hemoglobin (oxyhemoglobin + methemoglobin) from the scaffold over time, used as an indicator of blood component clearance. The sequential group exhibited delayed hemoglobin release compared with the mixed group, suggesting prolonged retention of blood-derived components within the scaffold. Data are presented as mean ± SD (n = 3).

**Fig. S5. Rheological characterization of neat PCL and blood-mixed PCL formulations.** (A) Temperature-sweep rheological analysis of neat liquid-type PCL and blood-mixed PCL formulations. Storage modulus (G’) and loss modulus (G”) were monitored with increasing temperature to evaluate the sol-to-network transition. The blood-mixed formulation showed a delayed G'-G'' crossover (gelation point) and attenuated development of G’ relative to neat PCL, indicating that blood admixture interferes with thermally induced network formation. (B) Frequency-sweep rheological analysis at 37 ℃. The neat PCL formulation exhibited consistently higher G′ values than the blood-mixed formulation across the tested frequency range, indicating a weaker, less elastic network in the blood-mixed condition.

**Fig. S6. *In vitro* mass variation of blood-PCL constructs and degradation behavior of blood-free PCL scaffolds.** (A) Mass variation (%) of mixed and sequential blood-PCL constructs was monitored over 240 h under in vitro conditions. Both groups showed an initial increase followed by a decline, with the sequential group exhibiting higher relative mass at later time points. Data are presented as mean ± SD (n = 3). (B) Remaining mass of blood-free PCL scaffolds during 10-day incubation under physiological conditions to evaluate the intrinsic degradation behavior of the PCL scaffold. **Inset**: GPC chromatograms after 0, 3, 5, 7, and 10 days of incubation, showing no pronounced shift in the main elution peak.

**Fig. S7. EDX elemental mapping and quantitative element composition of the mixed and sequential groups.** (A) Representative SEM images and corresponding EDX elemental maps of the mixed and sequential groups are shown. (B) Elemental distribution of C, O, Fe, and Ca were evaluated, and the corresponding quantitative compositions are summarized as weight percentages (wt%) in the table. The elemental compositions were normalized to the selected elements (C, O, Fe, and Ca). ND indicates that the element was not detected in the analyzed region under the measurement conditions. Ca in the mixed sample and Fe in the sequential sample were reported as ND, and maps for these channels are not shown.

**Fig. S8. Quantitative color analysis of in vivo bruise-like discoloration.** Longitudinal gross photographs were analyzed using an ImageJ-based color analysis workflow. Images were converted to the CIE L*a*b* color space, and the mean a* value was quantified within the injection/bruising site as a redness index reflecting blood-associated discoloration intensity. Sequential injection groups, particularly the blood-first group, showed more sustained redness intensity compared with the blood-only and mixed groups, indicating delayed resolution of visible discoloration.

**Fig. S9. CD86 and CD206 expression in tissue sections on days 5 and 10.** Representative immunofluorescence images and quantification of the CD206-positive/CD86-positive area ratio on day 5 (top) and day 10 (bottom). CD86, red; CD206, green; DAPI, blue. Groups 1–4 represent the control, Mixed, Blood→PCL, and PCL→Blood groups, respectively. Data are presented as mean ± SD, with n = 3 independent animals per group. Statistical comparisons among conditions were performed using one-way repeated-measures ANOVA followed by Tukey’s multiple-comparison test. Day 5 and Day 10 were analyzed separately. **p* < 0.05; ***p* < 0.01.

**Fig. S10. Representative Perls’ Prussian blue-stained images of injection-site tissues from each experimental group on day 10.** Low-magnification images are shown on the left, with dashed boxes indicating regions displayed at higher magnification on the right. Prussian blue-positive ferric iron deposits, consistent with hemosiderin accumulation, are indicated by red arrows. Data are presented as mean ± SD, with n = 3 independent animals per group. Statistical comparisons among conditions were performed using one-way repeated-measures ANOVA followed by Tukey’s multiple-comparison test. ****p* < 0.001.
